# 毛细胞白血病诊断与治疗中国指南（2023年版）

**DOI:** 10.3760/cma.j.issn.0253-2727.2023.12.001

**Published:** 2023-12

**Authors:** 中国抗癌协会血液肿瘤专业委员会 中华医学会血液学分会 中国慢性淋巴增殖性疾病工作组

毛细胞白血病（hairy cell leukemia，HCL）是一种少见的B细胞慢性淋巴增殖性疾病（B cell chronic lymphoproliferative diseases，B-CLPD），具有独特的临床、细胞/组织形态学、免疫表型及分子学特征[1]。随着对该病认识的深入及新药的应用，HCL的缓解率及生存得到极大改善。为提高我国HCL诊治水平，中国抗癌协会血液肿瘤专业委员会、中华医学会血液学分会及中国慢性淋巴增殖性疾病工作组组织专家根据国际相关指南及循证医学研究结果，结合我国诊治现状，制订了本指南，供相关医务工作者参考。

一、概述

HCL在欧美国家的年发病率约为2.9/100万人，中位发病年龄52岁，男女比例（2～4）∶1，亚洲人发病率明显低于欧美人[2]–[3]。该病临床表现为乏力、出血及反复感染等症状，常伴有全血细胞减少及脾大。其特征是骨髓、脾脏及外周血中“毛细胞”浸润，并具有典型的免疫表型，BRAF V600E突变是其标志性分子遗传学异常。

2022年世界卫生组织发布第5版造血与淋巴组织肿瘤分类（WHO-HAEM5）将HCL归类至脾B细胞淋巴瘤/白血病。过去通常将HCL分为经典型毛细胞白血病（classical hairy cell leukemia, cHCL）及变异型毛细胞白血病（hairy cell leukemia variant, HCL-v），然而，近年来研究认为HCL-v是一种不同于HCL的独立性疾病，具有独特的临床、免疫表型及分子学特征。因此，2022年WHO分型将HCL-v归类至伴有显著核仁的脾B细胞淋巴瘤/白血病（splenic B-cell lymphoma/leukemia with prominent nucleoli, SBLPN）[4]–[5]。本指南中HCL特指以往分类中的cHCL。

二、诊断、鉴别诊断及预后

（一）诊断

1. 临床表现：疲劳、乏力、出血、体重减轻等较常见，发热和盗汗较少见。约25％的HCL患者无症状。80％～90％的患者存在可触及的脾肿大，肝脏和淋巴结肿大者分别占20％和10％。60％～80％的患者表现为全血细胞减少，血小板、粒细胞减少者可出现出血、反复感染。仅10％～20％的患者外周血白细胞计数超过10×10^9^/L，多数患者伴有单核细胞减少（<0.1×10^9^/L），LDH水平正常[6]–[8]。

2. 外周血涂片：90％的HCL患者可见“毛细胞”。此类细胞中等大小，细胞核呈卵圆形、圆形或肾形，核仁不明显或缺如，染色质疏松，细胞核边界清晰，细胞质丰富，细胞膜周边具有毛状突起。

3. 骨髓细胞形态学及骨髓活检：由于骨髓纤维化及毛细胞的边缘互相交锁，骨髓常“干抽”。约10％的患者呈低增生骨髓象。骨髓中毛细胞呈弥散性或间质性浸润，极少见结节形式，少数病例可有窦内分布。骨髓活检往往显示中至重度的网状纤维增生，肿瘤细胞呈特征性“煎蛋”样形态。

4. 脾组织病理检查：显示红髓弥漫性扩张，白髓极度萎缩，毛细胞浸润、包围扩张的脾窦，形成“血湖”。

5. 免疫表型：HCL具有成熟B细胞表型，表达一种或多种免疫球蛋白重链，限制性表达κ或λ轻链。典型的免疫表型为CD5^−^，CD10^−^，CD19^+^，CD20^+^（bright），CD22^+^，CD11c^+^，CD25^+^，CD103^+^，CD123^+^，CD200^+^（bright），CD23^−^，cyclin D1^+^（灶性弱阳性），Annexin A1^+^[9]，CD27常阴性[10]。CD11c、CD25、CD103和CD123组合积分对HCL的诊断准确率高，98％的HCL患者积分在3～4分，而其他小B细胞淋巴瘤积分<3分[11]–[12]。耐酒石酸酸性磷酸酶（TRAP）染色阳性[13]。

6. 细胞遗传学异常：大约三分之二的HCL患者存在克隆性染色体异常，如5号染色体三体、倒位、缺失以及14号染色体三体、易位等[14]–[16]。

7. 分子遗传学特征：BRAF V600E突变存在于70％～100％的HCL患者中，是HCL的标志性遗传学异常[17]。此外，80％～90％的HCL患者存在免疫球蛋白重链可变区（IGHV）基因体细胞突变，IGHV无突变的患者常伴有白细胞增多，疾病更具侵袭性，且多伴有TP53突变，提示预后不良和原发性耐药[18]–[19]。10％～20％的HCL患者存在IGHV4-34重排，该类患者BRAF V600E突变率低，常伴有MAP2K1突变，其疾病进程类似HCL-v，对嘌呤类似物耐药，预后不良[20]。CDKN1B突变在HCL患者中也有报道[21]。

综上所述，HCL诊断主要依据临床表现、外周血及骨髓特征性细胞/组织形态学和典型免疫表型。对于免疫表型不典型的病例，应进行BRAF V600E突变及IGHV4-34重排检测，必要时检测MAP2K1、CDKN1B、IGHV、BCOR、KLF2、NOTCH2、CCND3等基因突变，以协助诊断。

（二）鉴别诊断

HCL的鉴别诊断主要参照《B细胞慢性淋巴增殖性疾病诊断与鉴别诊断中国专家共识（2018年版）》[22]，具体流程如图1所示，重点鉴别其他伴有脾肿大的B-CLPD，如SBLPN、脾弥漫性红髓小B细胞巴瘤/白血病（SDRPL）、脾边缘区淋巴瘤（SMZL）等[23]–[24]，鉴别要点详见表1。

**图1:**
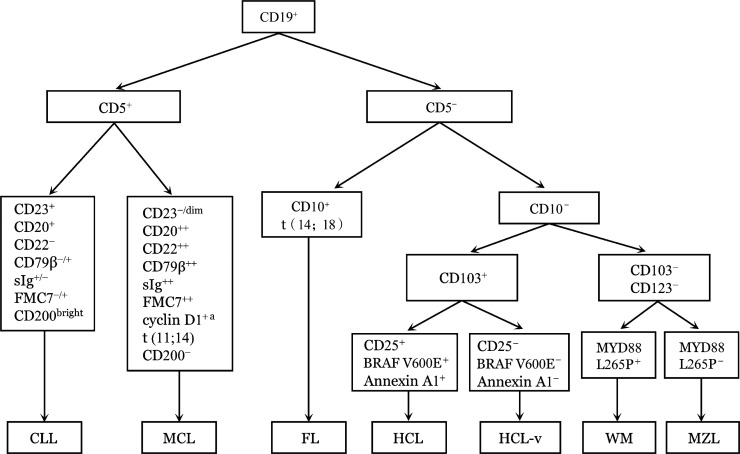
B细胞慢性淋巴增殖性疾病的免疫表型和细胞/分子遗传学鉴别诊断流程 注 ^a^ 免疫组织化学；CLL：慢性淋巴细胞白血病；MCL：套细胞淋巴瘤；FL：滤泡淋巴瘤；HCL：毛细胞白血病；HCL-v：变异型毛细胞白血病；WM：华氏巨球蛋白血症；MZL：边缘区淋巴瘤

**表1:** 脾B细胞淋巴瘤/白血病鉴别要点

临床检测项目	毛细胞白血病	伴有显著核仁的脾B细胞淋巴瘤/白血病	脾弥漫性红髓小B细胞淋巴瘤/白血病	脾边缘区淋巴瘤
外周血淋巴瘤细胞	细胞膜周边具有毛状突起	具有显著核仁，伴或不伴细胞质突起	细胞具有位于两极的细胞质突起，细胞质嗜碱性	细胞具有位于两极的短绒毛突起
免疫表型	CD20^+^(bright) CD103^+^ CD25^+^ CD27^−^ CD11c^+^ CD123^+^ Annexin A1^+^ cyclin D1^+^（灶性弱阳性）	CD20^+^(bright) CD103^+^ CD25^−^ CD27^+^ CD11c^+^ CD123^−^ Annexin A1^−^ cyclin D1^−^	CD20^+^(bright) CD103^−/+^ CD25^−^ CD27^+^ CD11c^−/+^ CD123^−^ Annexin A1^−^ cyclin D1^−^	CD20^+^ CD103^−^ CD25^−/+^ CD27^+^ CD11c^−/+^ CD123^−^ Annexin A1^−^ cyclin D1^−^
骨髓浸润	毛细胞呈弥散性或间质性浸润，极少见结节形式；多有中至重度网状纤维增多	间质性浸润，极少弥散，可侵犯髓窦；网状纤维增生不显著	窦内浸润、间质性浸润及结节形式均可见	结节形式及窦内浸润
脾脏病理	红髓弥漫性浸润形成“血湖”，白髓极度萎缩	红髓弥漫性浸润形成“血湖”，白髓极度萎缩	红髓弥漫性受累，白髓通常不累及	白髓显著扩张，红髓弥漫性浸润
细胞及分子遗传学	大部分患者具有BRAF V600E突变	无BRAF V600E突变，MAP2K1突变比例约42%	CCND3 PEST结构域突变	7q缺失（40%），NOTCH2及KLF2突变

（三）预后

HCL呈惰性病程，疾病进展缓慢。目前暂无统一的危险度分层体系，以下因素可能与不良预后相关：

1. 外周血白细胞计数>10×10^9^/L、毛细胞计数>5×10^9^/L、淋巴结肿大[19],[25]。

2. β_2_-微球蛋白>2倍正常值、LDH升高[26]–[27]。

3. 免疫表型表达CD38[28]。

4. 分子遗传学异常：IGHV4-34重排及MAP2K1突变常提示患者对嘌呤类似物耐药；而IGHV无突变及TP53突变常与不良预后及原发性耐药相关[18]–[19]。

三、治疗

（一）治疗指征

HCL患者诊断后并非立即需要治疗，当患者出现以下一种或多种情况时才需要启动治疗[12],[29]：

1. 全身症状（如发热、盗汗和不明原因的体重减轻等）；

2. 有症状的脾肿大或淋巴结肿大；

3. 反复感染；

4. 血细胞减少：中性粒细胞绝对计数<1×10^9^/L，血红蛋白<110 g/L，血小板计数<100×10^9^/L。

对于无症状或未达治疗指征的患者，应密切随访，建议每3～6个月随访1次，随访内容包括临床症状、体征和血常规等。

（二）治疗前评估

HCL患者治疗前（包括复发患者治疗前）应进行全面评估，至少包括：

1. 病史和体格检查（特别是浅表淋巴结受累区域和肝脾大小）；

2. 体能状态评分：如美国东部肿瘤协作组体能状态评分（ECOG评分）；

3. 实验室检查：血常规、肝肾功能、LDH、β_2_-微球蛋白、凝血功能；

4. HBV、HIV检测；

5. 外周血涂片检查；

6. 骨髓检查：包括细胞形态学、活检、免疫组化、流式免疫表型及染色体核型；

7. 影像学检查：推荐全身PET-CT或颈胸腹盆腔增强CT。

有条件的单位建议开展预后相关的分子生物学检测，包括IGHV突变及其片段使用、BRAF基因突变、TP53基因突变等以指导治疗。

（三）治疗方案

1. 初诊HCL的治疗方案：

（1）对于有治疗指征的患者，使用嘌呤类似物克拉屈滨±利妥昔单抗（Rituximab, R）作为初诊HCL的一线治疗方案。克拉屈滨治疗HCL完全缓解（CR）率高，中位缓解持续时间可达8年，常见的不良反应包括3～4级中性粒细胞减少、中性粒细胞缺乏伴发热、3～4级血小板减少和感染[30]–[31]。国外指南同时推荐另一种嘌呤类似物喷司他丁，但国内该药尚未上市。

克拉屈滨联合利妥昔单抗治疗初诊HCL患者CR率进一步提升，与延迟应用利妥昔单抗患者相比，两药同时应用的患者微小残留病（minimal residual disease, MRD）阴性率更高[32]–[33]。此外，具有治疗指征的HCL患者若伴有活动性感染，应注意先控制感染。

（2）BRAF抑制剂维莫非尼（Vemurafenib）不仅可治疗BRAF V600E突变的HCL，还可增加外周血白细胞，从而协助控制感染[34]。某些情况下（如极度虚弱、严重活动性感染、不能耐受嘌呤类似物治疗等）也可考虑应用维莫非尼±奥妥珠单抗治疗[29]。

（3）干扰素-α（IFN-α）曾是HCL的一线治疗药物，由于其耐受性差，且疗效明显低于嘌呤类似物，目前不推荐应用于HCL的一线治疗，仅在特殊情况下（如妊娠、严重感染、重度全血细胞减少等）作为初诊HCL的治疗药物[35]。

（4）脾切除是HCL的传统治疗方法，可以使约50％的患者血常规恢复正常，但不能达到长期缓解。因此，目前不推荐进行治疗性脾切除。

2. 疗效评估：初诊HCL患者应用初始方案治疗后，应进行疗效评估，从而指导下一步治疗及评估预后。评估时机如下：

接受克拉屈滨治疗的患者，应在治疗结束至少4～6个月后进行疗效评估；若疗效为部分缓解（PR），可选择在治疗结束至少6个月后给予第2个疗程的克拉屈滨±利妥昔单抗治疗[36]。HCL疗效评估标准见表2[6]。

**表2:** 毛细胞白血病疗效评估标准

疗效	评估指标
完全缓解（CR）	外周血细胞计数接近正常：血红蛋白>110 g/L（未输血）；血小板计数>100×10^9^/L；中性粒细胞绝对计数>1×10^9^/L。体格检查显示脾肿大消失。外周血涂片和骨髓检查中均未找到HCL的形态学证据
CR伴或不伴MRD	对于获得CR的患者，应用骨髓流式细胞术或免疫组织化学评估MRD
部分缓解（PR）	外周血细胞计数接近正常（同CR），肿大器官缩小及骨髓浸润减少≥50%
CRu或PRu	未复查骨髓，但符合CR或PR的其他标准
疾病稳定（SD）	治疗后未达到以上缓解标准及PD标准的患者
疾病进展（PD）	疾病相关症状加重，肿大器官增大或血细胞计数下降≥25%，排除治疗后骨髓抑制引起的血细胞计数下降
HCL复发	血液学复发是指再次出现低于上述CR和PR标准的血细胞减少。形态学复发是指在血液学未复发的情况下，外周血和（或）骨髓中再次出现毛细胞

注 HCL：毛细胞白血病；MRD：微小残留病

目前MRD阴性HCL患者的生存是否延长尚没有确切结论，MRD对预后的影响仍需要更多的临床研究来证实。

3. 复发HCL患者的治疗方案：尽管HCL初始治疗的缓解率高，缓解持续时间长，但仍有50％左右患者复发。复发时应首先确认诊断的准确性，并明确是否存在不良预后因素（例如严重贫血、脾脏位于左肋缘下>10 cm、免疫表型异常、BRAF V600E突变缺失等）。再治疗前也需要评估是否具有治疗指征（同初治）。

（1）对于复发前缓解时间≥2年的患者，推荐克拉屈滨＋利妥昔单抗重新治疗。复发后再次应用克拉屈滨单药治疗可能导致反应率下降，缓解期缩短，而联合利妥昔单抗治疗复发HCL患者的CR率提高，生存显著延长[32]–[33],[37]。

（2）对于复发前缓解时间<2年的患者，在确认诊断准确后，推荐临床试验，或考虑应用BRAF抑制剂维莫非尼±利妥昔单抗治疗。维莫非尼单药在复发/难治HCL患者中总缓解率（ORR）高，但CR率偏低[38]。维莫非尼联合利妥昔单抗对大部分复发/难治HCL患者有效，CR率较高[39]。一项小样本研究显示，苯达莫司汀＋利妥昔单抗治疗复发HCL具有良好疗效[40]。此外，聚乙二醇干扰素α-2a（PEG-IFNα-2a）治疗也具有一定获益[41]。

（3）对于一线治疗后未达CR的HCL患者，治疗方案参照缓解时间<2年复发患者的治疗方案。体能状态差或老年患者达到PR后，可暂停治疗，密切监测，待疾病进展时再启动治疗。

4. ≥2线治疗的复发/难治HCL患者的治疗方案：首选推荐进入临床试验，如果没有合适的临床试验，也可应用CD22抗体-免疫毒素偶联物莫塞妥莫单抗（Moxetumomab pasudotox）治疗。治疗时应注意监测并预防毛细血管渗漏综合征（CLS）和溶血尿毒综合征（HUS）[42]。未应用过维莫非尼±利妥昔单抗治疗的患者也可采用该治疗方案。

此外，BTK抑制剂（BTKi）也对部分患者有效。最常见的≥3级不良反应为贫血、血小板减少及中性粒细胞减少[43]。

上述具体方案及流程见表3、图2。

**表3:** 毛细胞白血病治疗方案

患者类型	首选方案	其他方案
初诊毛细胞白血病患者	•克拉屈滨^a^±利妥昔单抗^b^	•干扰素-α^e^（妊娠、严重感染、重度全血细胞减少患者） •维莫非尼±奥妥珠单抗^f^（嘌呤类似物不耐受、严重感染、极度虚弱患者）
初始治疗后未达CR或CR<2年复发患者	•临床试验 •维莫非尼^c^±利妥昔单抗	•苯达莫司汀＋利妥昔单抗 •干扰素-α/聚乙二醇干扰素α-2a •利妥昔单抗（无法应用嘌呤类似物治疗的患者）
CR≥2年复发患者	•克拉屈滨＋利妥昔单抗	•利妥昔单抗（无法应用嘌呤类似物治疗的患者）
≥2线治疗的复发/难治患者	•临床试验 •莫塞妥莫单抗^d^ •维莫非尼±利妥昔单抗（前期未应用的患者）	•BTKi ^g^[43]

注 ^a^克拉屈滨：0.10 mg·kg^−1^·d^−1^，持续静脉输注×7 d；或0.14 mg·kg^−1^·d^−1^，静脉输注2 h×5 d；或0.10～0.14 mg·kg^−1^·d^−1^，皮下注射×5 d[6]。^b^利妥昔单抗：375 mg·m^−2^/周×6 ~ 8周，每周1次，静脉输注。^c^维莫非尼：960 mg，口服，每日2次。^d^莫塞妥莫单抗：0.04 mg/kg，第1、3、5天静脉给药，28 d为1个周期，持续6个周期或直至疾病进展或出现不可接受的毒性。^e^干扰素-α：3×10^6^ U每日皮下注射至最大反应，此后每周3次维持治疗。^f^奥妥珠单抗：1000 mg，静脉输注，治疗第2个月第1、8和15天，第3个月第1天，第4个月第1天用药。^g^ BTKi：伊布替尼：420 mg，口服，每日1次，直至疾病进展或出现不可接受的毒性。CR：完全缓解

**图2:**
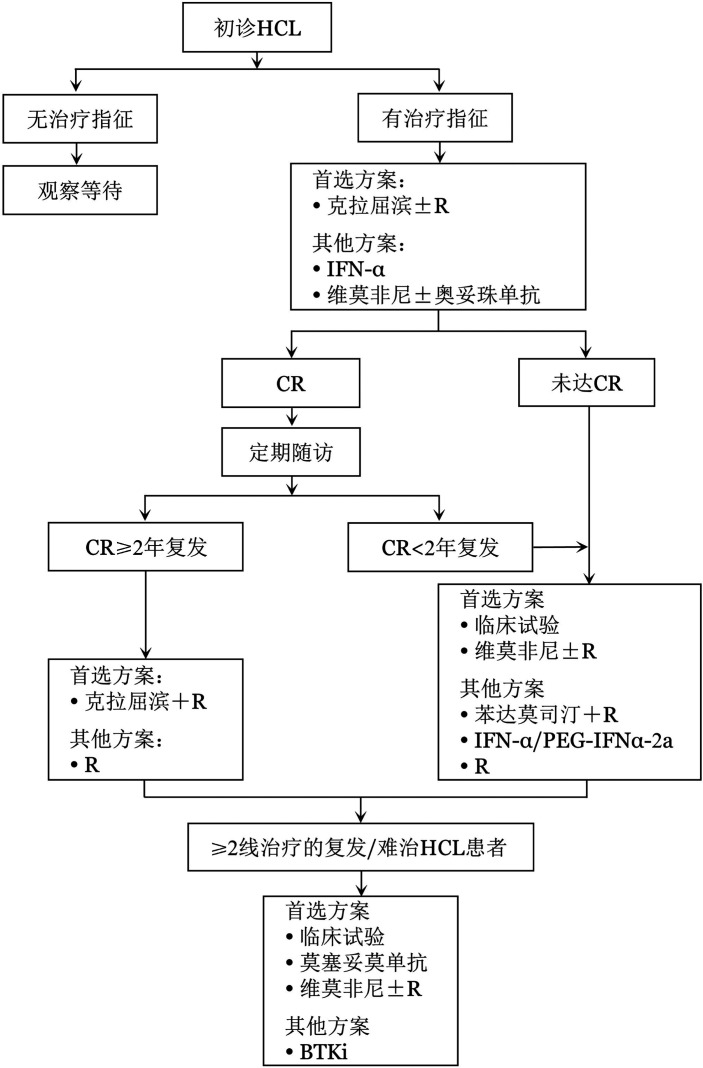
毛细胞白血病患者推荐治疗流程图 注 HCL：毛细胞白血病；R：利妥昔单抗；IFN-α：干扰素-α；CR：完全缓解；PEG-IFNα-2a：聚乙二醇干扰素α-2a；BTKi：BTK抑制剂

5. SBLPN（含HCL-v）的治疗：SBLPN包括既往WHO分型中的HCL-v、部分B细胞幼淋巴细胞白血病等。目前SBLPN没有公认的最佳治疗方案。以往对于具有治疗指征的初诊HCL-v患者，可考虑采用克拉屈滨/苯达莫司汀＋利妥昔单抗作为一线治疗方案[44]–[45]。对于复发/难治HCL-v，推荐进入临床试验。小样本队列研究显示伊布替尼、莫塞妥莫单抗对复发/难治HCL-v有效[46]–[47]。

6. 支持治疗：

（1）感染的预防及处理：嘌呤类似物治疗容易引起感染并发症，应注意防治[29]。中性粒细胞减少患者参照《中性粒细胞减少症诊治中国专家共识》进行预防及治疗[48]。具有治疗指征的HCL患者若合并新型冠状病毒感染（COVID-19），应根据疾病进展程度及治疗相关免疫抑制不良反应等因素进行治疗选择，详见《惰性B细胞非霍奇金淋巴瘤患者新型冠状病毒感染防治专家共识》[49]。BRAF V600E突变的HCL患者合并中性粒细胞减少及COVID-19时，可应用BRAF抑制剂治疗，以控制病情并减轻免疫抑制[50]。

（2）HBV再激活：建议所有接受免疫治疗和（或）化疗的HCL患者参照《淋巴瘤免疫化疗乙型肝炎病毒再激活预防和治疗中国专家共识》进行HBV的预防及治疗[51]。

（3）输血：嘌呤类似物治疗后可引起骨髓抑制，应密切监测血象。对于需要输血治疗的患者，建议给予辐照红细胞和（或）血小板输注。

四、随访

治疗完成后的前2年应每3个月进行一次随访，第3～5年每半年进行一次随访，5年后每1年进行一次随访。随访内容包括临床症状、肝脾淋巴结查体、血细胞计数及生化检查等。此外，HCL患者继发性恶性肿瘤的发生率增加，约10％的患者继发血液系统恶性肿瘤和实体瘤，尤其是骨髓瘤、霍奇金淋巴瘤、非霍奇金淋巴瘤、黑色素瘤和甲状腺癌，需注意及时鉴别及治疗[52]。
